# Comparative study on the sensitivity of lumbar puncture and external ventricular drainage cerebrospinal fluid in the diagnosis of central nervous system infections after craniotomy

**DOI:** 10.3389/fneur.2025.1586394

**Published:** 2025-04-15

**Authors:** Yue Tang, Hongmiao Xu, Dong Liu, Tingting Lin, Fujun Zuo, Dingli Wen, Yinjuan Liao, Zhennan Ye, Peng Liu, Jincan Zhang

**Affiliations:** ^1^Department of Neurosurgery, The Fourth Hospital of Changsha, Changsha, China; ^2^Department of Critical Care Medicine, The Fourth Hospital of Changsha, Changsha, China; ^3^Department of Clinical Pharmacy, The Fourth Hospital of Changsha, Changsha, China; ^4^Department of Neurosurgery, The Second Affiliated Hospital of Guangzhou Medical University, Guangzhou, China; ^5^Department of Neurosurgery, Peking University Third Hospital, Beijing, China

**Keywords:** lumbar puncture, external ventricular drainage, cerebrospinal fluid, central nervous system infections, craniotomy

## Abstract

Early and accurate diagnosis of central nervous system infections (CNSIs) is crucial for clinical treatment. Cerebrospinal fluid (CSF) analysis is considered the gold standard for diagnosis, and the sensitivity of its results directly affects treatment decisions. However, due to physiological differences in CSF composition between the ventricles and the lumbar space, the choice of sampling site may lead to diagnostic discrepancies, including false negatives. This study aims to compare the diagnostic sensitivity of CSF samples obtained via external ventricular drainage (EVD) and lumbar puncture (LP) in patients with CNSIs following craniotomy.

**Methods:**

This study prospectively collected data from patients who underwent craniotomy and had EVD placement between January 2024 and December 2024. For patients suspected of CNSIs, CSF samples were simultaneously collected via LP and EVD, and the differences in cell counts and biochemical markers were compared. The Kappa index was used to assess diagnostic sensitivity and correlation, and statistical analysis was performed using McNemar’s χ^2^ test.

**Results:**

A total of 41 patients were included, with 41 LP samples and 41 EVD samples collected. Among the 82 samples, 29 met the diagnostic criteria for CNSIs, with 21 (72.4%) from LP samples and 8 (27.6%) from EVD samples. Among the 21 LP-diagnosed infection cases, 14 EVD samples did not meet the infection criteria, while among the 8 EVD-diagnosed infection cases, only 1 LP sample did not meet the infection criteria. The Kappa correlation index between LP and EVD diagnostic results was 0.279, and McNemar’s χ^2^ test yielded *p* = 0.001.

**Conclusion:**

LP CSF demonstrates higher sensitivity than EVD CSF for early diagnosis of CNSIs in post-craniotomy patients with indwelling EVDs. In clinical practice, when EVD results are negative but there is high clinical suspicion of CNSIs, concurrent LP should be performed for further confirmation.

## Background

Central nervous system infections (CNSIs) are life-threatening infectious diseases with a mortality rate of 15%–30% (1–3). Early identification and timely treatment are crucial for improving patient outcomes. In neurosurgery, craniotomy combined with external ventricular drainage (EVD) is widely used for treating aneurysmal subarachnoid hemorrhage (aSAH), deep brain hematomas, and brain tumors. However, craniotomy itself carries a risk of CNSIs (4), and EVD placement further increases this risk (5, 6). For patients with EVD, CSF is typically collected from the EVD when CNSIs are suspected.

It is noteworthy that although CSF analysis is considered the gold standard for diagnosing CNSIs, recent studies have reported cases where ventricular CSF was normal while lumbar CSF showed significant abnormalities in CNSIs patients (7, 8). This suggests that the choice of CSF sampling site may significantly impact the diagnosis of CNSIs. Currently, there is a lack of systematic research data on the differences between LP and EVD CSF analysis in monitoring and treating CNSIs in patients who have undergone craniotomy with EVD placement.

Based on this, this study aims to explore the differences in diagnostic sensitivity between EVD and LP CSF samples in patients with CNSIs after craniotomy with EVD placement, providing clinicians with more reliable diagnostic evidence.

## Methods

We prospectively collected data from a single treatment group of neurosurgical patients who underwent craniotomy between January 2024 and December 2024.

### Inclusion criteria

Patients who underwent craniotomy with EVD placement and developed fever with elevated peripheral blood infection markers during prophylactic antibiotic treatment. Exclusion criteria included: (1) patients with brain abscess or open head injury; (2) patients with a previous diagnosis of intracranial infection; (3) patients with contraindications to LP.

### Sample collection and processing

All samples were collected by the first author under strict aseptic conditions. The collection sequence was as follows: first, EVD CSF samples were collected, followed by clamping the drainage tube and performing LP within 30 min to obtain CSF samples. Both sets of samples were clearly labeled to ensure no confusion during laboratory analysis.

### Data collection

The collected indicators included: (1) Demographic data: gender, age; (2) Clinical data: primary disease, time points of specimen collection; (3) Microbiological results: Gram staining, culture results; (4) CSF indicators: white blood cell (WBC) count, glucose level, chloride level, protein level, lactate dehydrogenase (LDH) level, and random blood glucose at the time of collection.

### Diagnostic criteria for CNSIs

(1) Body temperature > 38°C with altered mental status, CSF WBC count > 100 × 10^6^/L, neutrophil ratio > 70%, protein concentration > 0.45 g/L, CSF glucose/serum glucose ratio < 0.4; (2) Positive CSF microbial culture.

The clinical diagnosis of CNSIs is established when criterion (1) is met, while the etiological diagnosis of CNSIs requires fulfillment of both (1) and (2).

Statistical analysis was performed using SPSS 26.0 software (SPSS Inc., Chicago, Illinois, United States). Categorical variables were expressed as numbers and percentages. Numerical variables were assessed for normality using the Kolmogorov–Smirnov test. Normally distributed variables were expressed as mean and standard deviation, while non-normally distributed variables were expressed as median and interquartile range. The Kappa concordance index was used to analyze the correlation between EVD and LP results, and McNemar’s χ^2^ test was used for validation. For normally distributed data, paired t-tests were used; for non-normally distributed data, Mann–Whitney U tests or Wilcoxon Signed-Rank tests were used. A *p*-value <0.05 was considered statistically significant.

## Results

Between January 2024 and December 2024, 387 patients underwent neurosurgical craniotomy in our group. Ultimately, 41 patients met the inclusion criteria, including 4 with aSAH (aneurysmal subarachnoid hemorrhage), 26 with deep brain hematoma, 4 with intracranial tumor, 2 with AVM (arteriovenous malformations), and 5 with traumatic brain injury. A total of 82 CSF samples were collected (41 from EVD and 41 from LP). The average time from craniotomy to CSF sample collection was 140.1 ± 43.7 h, and detailed clinical data are shown in Table 1.

**Table 1:** Baseline characteristics of patients.

Characteristics	Value
No. of patients	41
Age, y	57.2 ± 17.5
Sex, *N* (%)	
Male	29 (70.7%)
Female	12 (29.3%)
CSF collection time to craniotomy, h	140.1 ± 43.7
Primary brain pathology, *N* (%)	
aSAH	4 (9.8%)
Deep brain hematoma	26 (63.4%)
Intracranial tumor	4 (9.8%)
AVM	2 (4.9%)
Traumatic brain injury	5 (12.1%)

CSF, cerebrospinal fluid; aSAH, aneurysmal subarachnoid hemorrhage; AVM, arteriovenous malformation.

**Figure 1:**
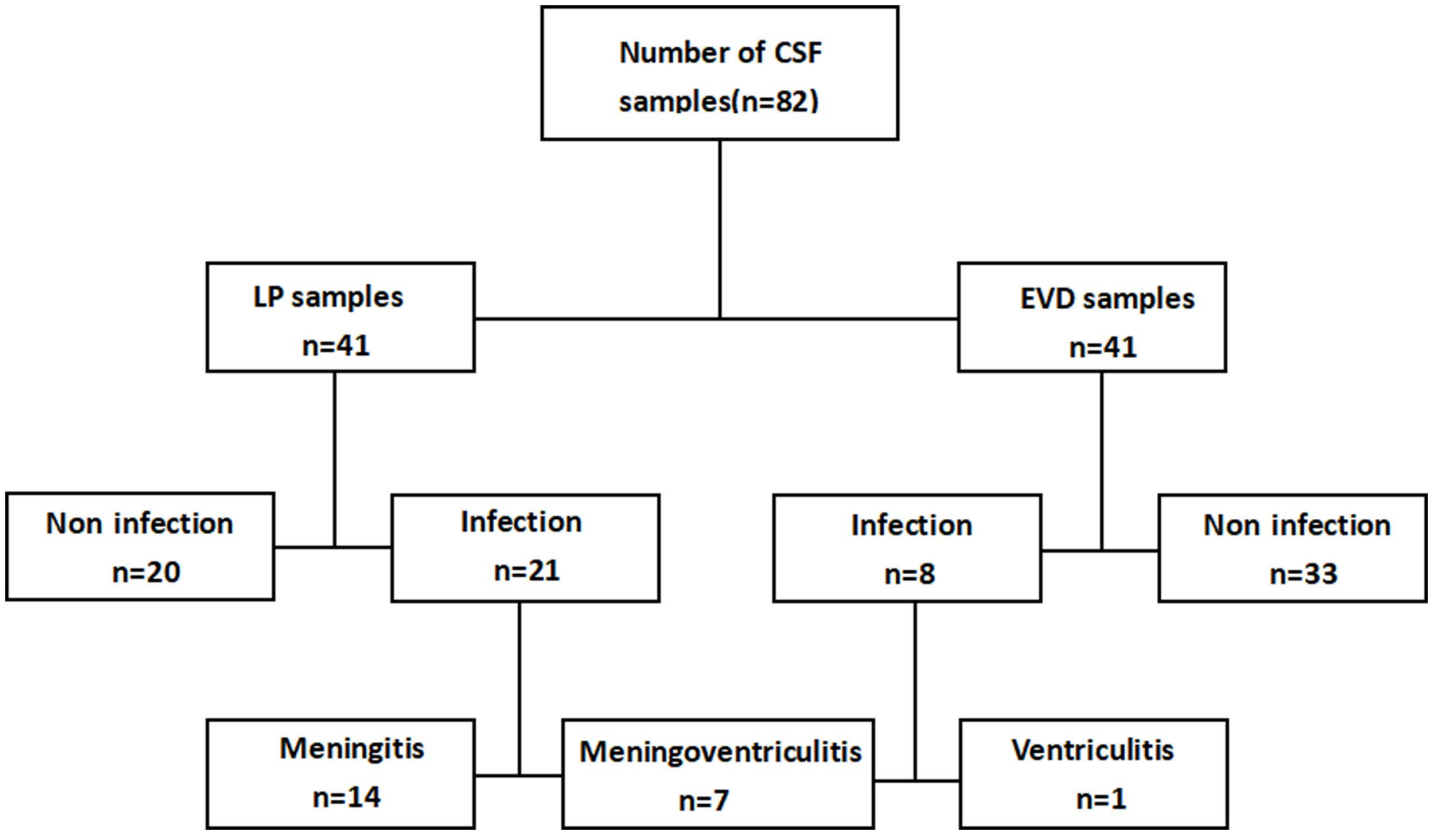
Sample results diagram.

Among the 82 samples, 29 met the CNSIs criteria. Separate analysis showed that 21 of the 41 LP samples met the CNSIs criteria, while only 8 of the 41 EVD samples met the criteria. Among the 21 LP samples that met the infection criteria, 14 EVD samples did not meet the criteria. Among the 8 EVD samples that met the infection criteria, only 1 LP sample did not meet the criteria (Figure 1). The Kappa correlation index between LP and EVD infection diagnostic results was 0.279 (weak correlation), and McNemar’s χ^2^ test further confirmed the weak correlation (*p* = 0.001, Table 2).

**Table 2:** Comparison of the incidence of CNSIs between LP and EVD.

Groups	*n*	Number with CNSIs of EVD	Number without CNSIs of EVD
Number with CNSIs of LP	21	7	14
Number without CNSIs of LP	20	1	19
Kappa correlation index			0.279
McNemar χ^2^			0.001

CNSIs, central nervous system infections; LP, lumbar puncture; EVD, external ventricular drainage.

The 41 patients were divided into four groups: non-CNSIs group (19 cases), LP infection + EVD non-infection group (14 cases), LP infection + EVD infection group (7 cases), and LP non-infection + EVD infection group (1 case). The differences in CSF biochemical markers (WBC count, glucose, chloride, protein, LDH levels) were analyzed among the groups. The sample collection time for the CNSIs and non-CNSIs groups was 138.8 ± 50.7 h and 141.7 ± 35.2 h, respectively, with no statistical difference (*p* = 0.8316).

**Figure 2:**
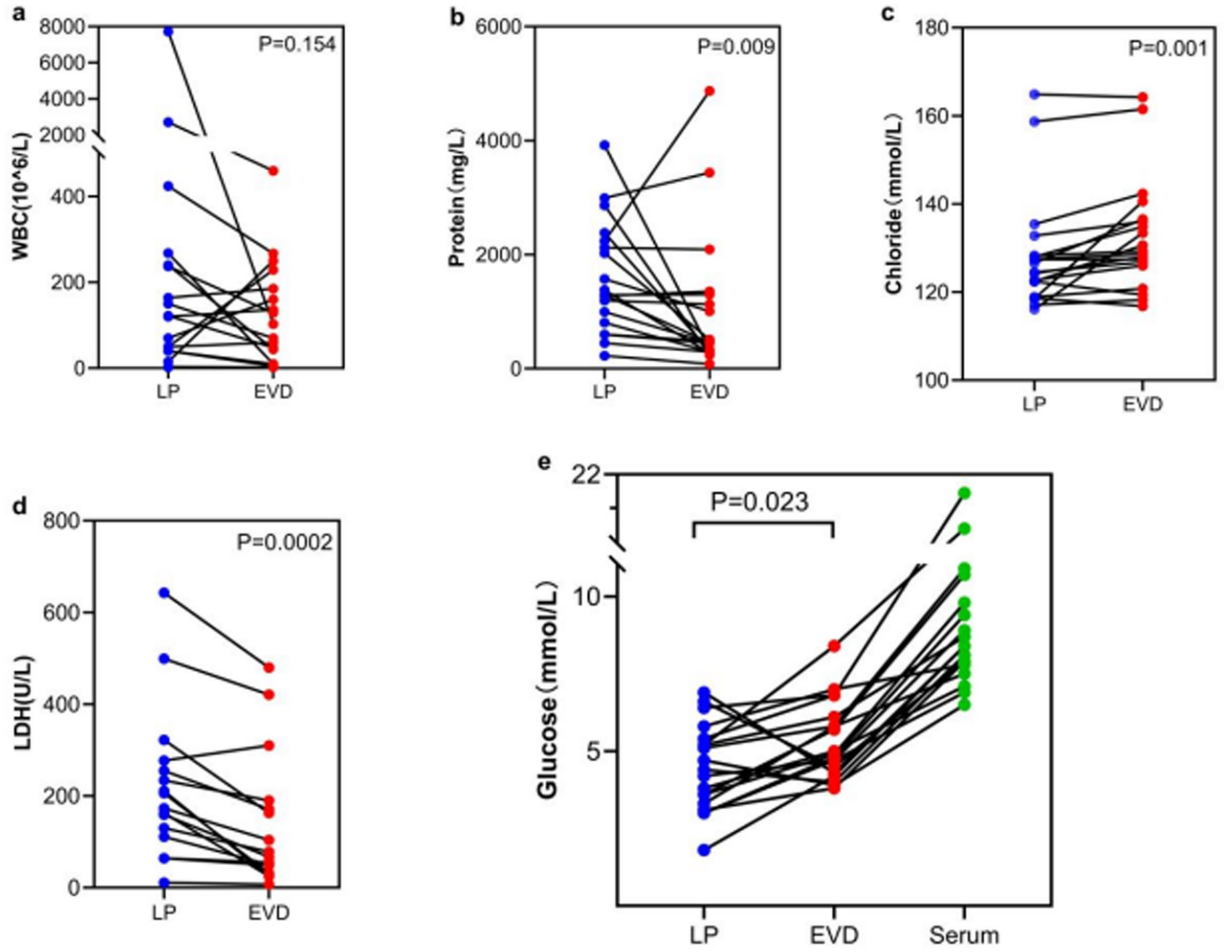
Comparison of CSF indicators between LP and EVD groups in the Non-CNSIs group. **(a)** WBC-Median: LP 120[20–240] vs. EVD 70[10–185], *p* = 0.154; **(b)** Protein-Average: LP 1590.4 ± 974.7 vs. EVD 1019.8 ± 1238.2, *p* = 0.009; **(c)** Chloride-Average: LP 128.2 ± 13.0 vs. EVD 132.3 ± 12.8, *p* = 0.001; **(d)** LDH-Median: LP 255[206–499] vs. EVD 310[163–421], *p* = 0.0002 **(e)** Glucose-Average: LP 4.5 ± 1.4 vs. EVD 5.3 ± 1.3, *p* = 0.023.

In the non-CNSIs group, LP WBC counts were higher than EVD, but not statistically significant (120[40–240] vs. 70[10–185], *p* = 0.154), while LP glucose and chloride levels were significantly lower than EVD (4.5 ± 1.4 vs. 5.3 ± 1.3, *p* = 0.023, 128.2 ± 13.0 vs. 132.3 ± 12.8, *p* = 0.001), and LP protein and LDH levels were significantly higher than EVD (1590.4 ± 974.7 vs.1019.8 ± 1238.2, *p* = 0.009; 255[206–499] vs. 310[163–421], *p* = 0.0002) (Figure 2).

**Figure 3:**
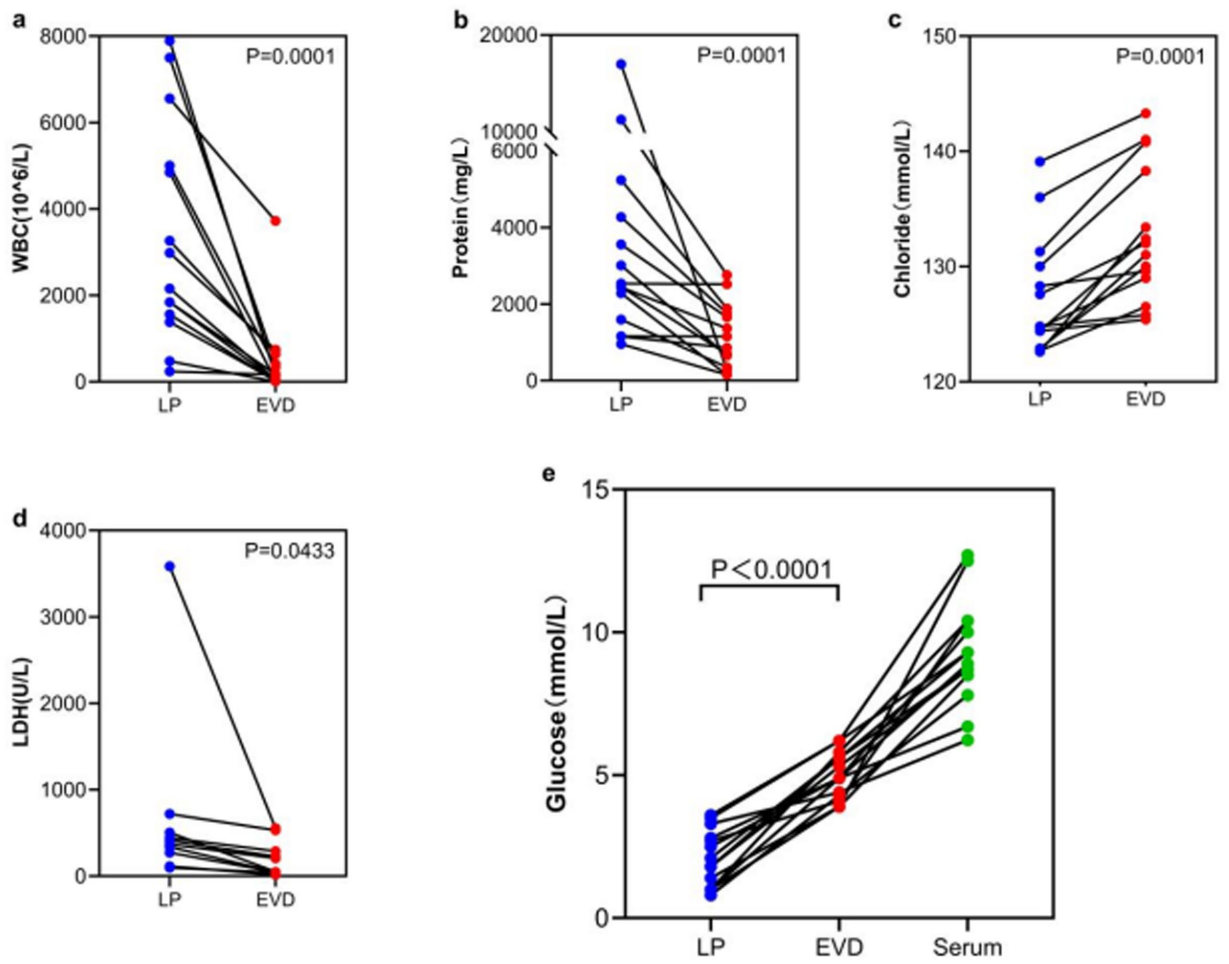
Comparison of CSF indicators between LP infection and EVD non-infection group. **(a)** WBC-Average: LP 3396 ± 2,544 vs. EVD 462 ± 969, *p* = 0.0001; **(b)** Protein-Average: LP 4210.2 ± 4512.2 vs. EVD 1161.0 ± 861.3, p = 0.0001; **(c)** Chloride-Average: LP 127.3 ± 5.2 vs. EVD 132.8 ± 5.9, p = 0.0001; **(d)** LDH-Median: LP 3585 [443–3,585] vs. EVD 531[229–556], *p* = 0.0433; **(e)** Glucose-Average: LP 2.1 ± 1.0 vs. EVD 5.0 ± 0.8, *p* < 0.0001.

In the LP infection + EVD non-infection group, LP WBC counts, LDH, and protein levels were significantly higher than EVD (3,396 ± 2,544 vs. 462 ± 969, *p* = 0.0001; 3,585[443-3585]vs.531[229–556], *p* = 0.0433; 4210.2 ± 4512.2 vs. 1161.0 ± 861.3, *p* = 0.0001), while LP glucose and chloride levels were significantly lower than EVD (2.1 ± 1.0 vs. 5.0 ± 0.8, *p* < 0.0001; 127.3 ± 5.2 vs. 132.8 ± 5.9, *p* = 0.0001) (Figure 3).

**Figure 4:**
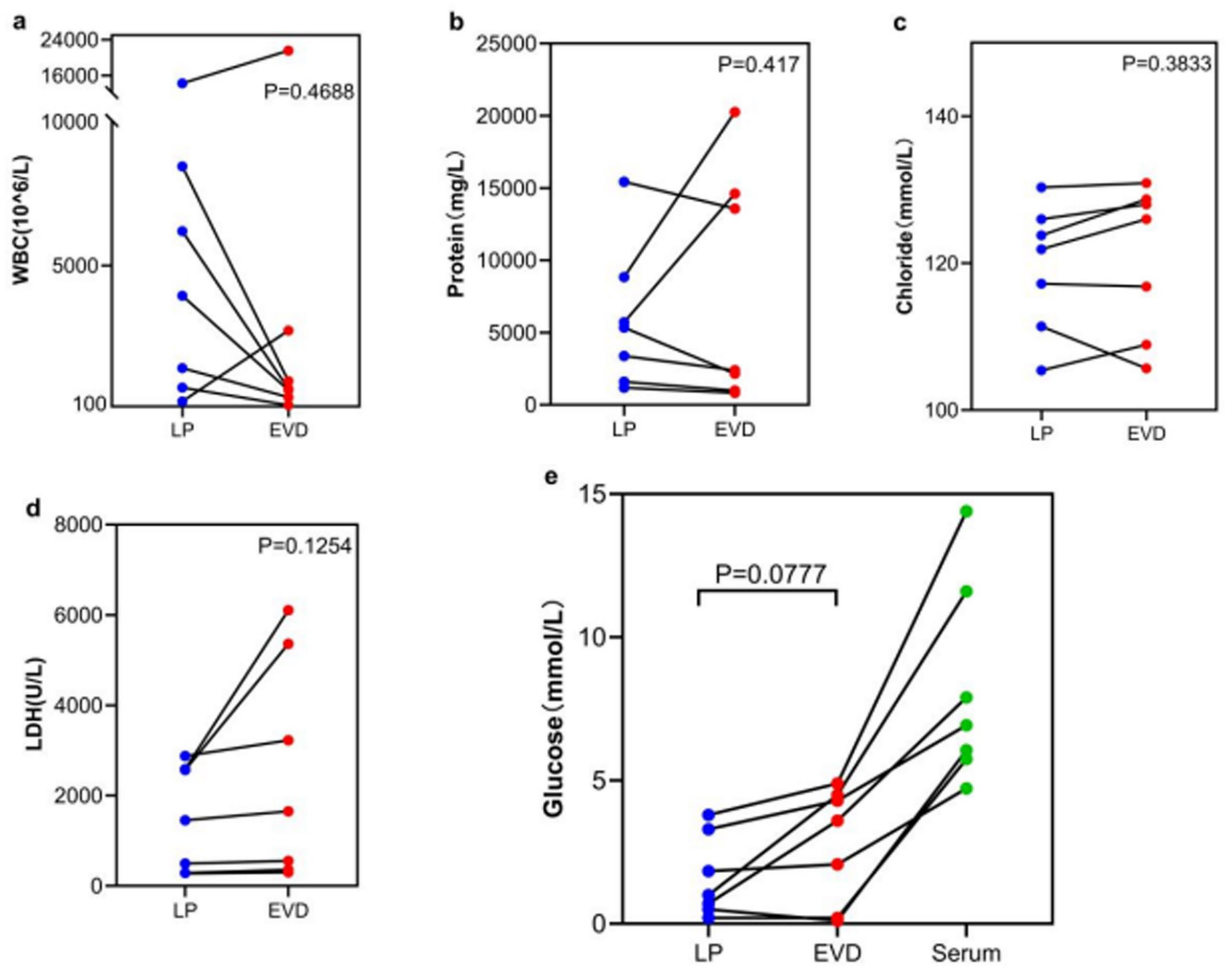
Comparison of CSF indicators between LP infection and EVD infection group. **(a)** WBC-Average: LP 5054 ± 5,065 vs. EVD 3892 ± 7,832, *p* = 0.4688; **(b)** Protein-Average: LP 5929.3 ± 4947.7 vs. EVD 7828.7 ± 8076.9, *p* = 0.417; **(c)** Chloride-Average: LP 119.4 ± 8.7 vs. EVD 120.7 ± 10.2, *p* = 0.3833; **(d)** LDH-Average: LP 1505.7 ± 1168.7 vs. EVD 2507.9 ± 2436.1, *p* = 0.1254; **(e)** Glucose-Average: LP 1.6 ± 1.4 vs. EVD 2.8 ± 2.0, *p* = 0.0777.

In the LP infection + EVD infection group, LP WBC counts were higher than EVD, but not statistically significant (5,054 ± 5,065 vs. 3,892 ± 7,832, *p* = 0.4688). LP glucose, chloride, LDH, and protein levels were lower than EVD, but not statistically significant (1.6 ± 1.4 vs. 2.8 ± 2.0, *p* = 0.0777; 119.4 ± 8.7 vs. 120.7 ± 10.2, *p* = 0.3833; 1505.7 ± 1168.7 vs. 2507.9 ± 2436.1, *p* = 0.1254; 5929.3 ± 4947.7 vs. 7828.7 ± 8076.9, *p* = 0.417) (Figure 4).

In the one case of EVD infection + LP non-infection, EVD and LP WBC counts were 430 × 10^6^/L and 760 × 10^6^/L, respectively. EVD and LP glucose levels were 3.7 and 6.9 mmol/L (random blood glucose was 12.5 mmol/L). EVD and LP chloride levels were 119.2 and 125.3 mmol/L. EVD and LP protein levels were 972.2 mg/L and 185.8 mg/L, and EVD and LP LDH levels were 167 and 32 U/L.

Among the 29 samples that met the CNSIs criteria, only 1 (3.4%) LP sample detected a pathogen, which was *Ureaplasma parvum*.

## Discussion

In clinical practice, early diagnosis of CNSIs is challenging for neurosurgeons because neurological signs and symptoms are often nonspecific. The diagnosis of suspected CNSIs heavily relies on CSF analysis. When obtaining CSF for diagnostic purposes, clinicians can choose between LP or EVD. However, in patients with EVD, collecting CSF from the EVD is generally preferred over repeated LP for ease and patient comfort. Due to physiological differences in CSF composition between the ventricles and the lumbar space (9–11), the choice of sampling site may lead to diagnostic discrepancies, including false negatives, complicating the diagnosis. Recent studies have reported cases where ventricular CSF was normal while lumbar CSF showed significant abnormalities in CNSIs patients (7, 8), suggesting that the choice of CSF sampling site may significantly impact the sensitivity of CNSIs diagnosis. Currently, there is limited data on the relationship between CSF sampling site and diagnostic sensitivity in patients with EVD placement after craniotomy, prompting us to conduct this study.

This study compared the biochemical and microbiological indicators of LP and EVD CSF samples in patients with EVD placement after craniotomy. Among the 82 samples, 29 met the CNSIs criteria, with 21 (72.4%) detected in LP CSF samples and only 8 (27.6%) detected in EVD CSF samples. LP CSF demonstrated higher sensitivity in diagnosing CNSIs. Kakadia et al. (7) reported in a case series that LP CSF had significantly higher WBC counts and protein concentrations than EVD samples, and EVD samples may underestimate infection severity due to craniocaudal gradient and dilution effects, leading to “pseudo-sterilization.” In this study, the infection detection rate for LP samples was 51.2% (21/41), while for EVD samples it was only 19.5% (8/41), and the Kappa concordance index was only 0.279. McNemar’s χ^2^ test (*p* = 0.001) further confirmed the high heterogeneity between the two sampling sites. Finger et al. (11) also noted in a prospective study that LP sensitivity (90%) was significantly higher than EVD (41.5%), and LP could independently diagnose 58.4% of infection cases. Our results also support that relying solely on EVD samples may lead to missed diagnoses or delayed treatment.

The differences between LP and EVD samples may stem from the following mechanisms:

1. Craniocaudal Biochemical Gradient: Under physiological conditions, CSF is produced by the choroid plexus in the ventricles and flows unidirectionally to the spinal subarachnoid space, where it is reabsorbed. This CSF flow results in higher cell counts and protein concentrations in the lumbar CSF compared to the ventricular CSF (12–14). This study found that in the non-CNSIs group, glucose (*p* = 0.023) and chloride (*p* = 0.001) levels in LP CSF were significantly lower than in EVD samples, while protein (*p* = 0.009) and LDH (*p* = 0.0002) levels were significantly higher, confirming the existence of a baseline gradient effect. Notably, this physiological gradient may introduce diagnostic bias in LP samples: elevated WBC counts and protein levels, along with decreased glucose and chloride levels, could potentially push some actually sterile samples to meet laboratory diagnostic thresholds for CNSIs. This is particularly relevant in the LP infection but EVD non-infection subgroup, necessitating cautious clinical interpretation of these parameters. Although the CNSIs group did demonstrate more pronounced gradient differences (WBC *p* = 0.0001, protein *p* = 0.0001, LDH *p* = 0.0433, glucose *p* < 0.0001, and chloride *p* = 0.0001), it must be emphasized that relying solely on routine biochemical indicators from LP samples may not reliably distinguish between infectious inflammation and gradient amplification effects. The current findings suggest that in post-craniotomy patients, traditional CSF test parameters may be more significantly affected by craniocaudal gradient interference compared to the general population, providing a potential biological basis for false-positive diagnoses. Future approaches may require combining novel specific biomarkers with gradient correction algorithms to more accurately differentiate true CNSIs from gradient-related false positives.
2. Infection Spread Pattern: After craniotomy, the blood–brain barrier is disrupted to varying degrees. When infection involves the meninges, the inflammatory response is initially triggered locally in the meninges, leading to a significant increase in inflammatory markers (such as WBC, protein, and LDH) in the lumbar CSF, followed by lower glucose concentrations, and then spreading to the ventricles (15, 16). However, ventricular CSF, being farther from the inflammatory center and affected by dilution, may show “false-negative” results. In this study, the 14 cases with LP infection but EVD non-infection may represent an early stage of meningitis, suggesting that LP CSF might be more sensitive in detecting early CNSIs. In the subgroup with both LP and EVD infection (*n* = 8), there were no significant differences in WBC, glucose, chloride, LDH, or protein levels between the two sample types (all *p* > 0.05). For infections involving both the lumbar and ventricular spaces, both EVD and LP samples can provide reliable diagnostic information. The single case of EVD infection but LP non-infection might indicate localized ventriculitis. This observation aligns with the “unidirectional cerebrospinal fluid flow-mediated infection spread” hypothesis proposed by Finger et al. (11).

The results of this study emphasize that LP should be the “first choice” for diagnosing CNSIs in patients with EVD placement after craniotomy. Therefore, in clinical practice, when EVD samples are negative but CNSIs are highly suspected, LP should be performed as early as possible to rule out false negatives. Additionally, during antibiotic treatment, repeated sampling from the same site (preferably LP) is recommended to assess treatment efficacy and avoid misjudgment due to gradient differences.

Nevertheless, this study still has some limitations: (1) This study included only 41 patients, with a small sample size and single-center design, which may limit the generalizability of the results.(2) Although there was no statistical difference in sample collection time between the CNSIs and non-CNSIs groups, the time from surgery to sampling (average 140 h) may affect the intensity of the inflammatory response, requiring larger samples for validation.(3) The low pathogen detection rate (only 3.4%) may be related to antibiotic pretreatment or limitations in culture techniques. Future studies should incorporate high-sensitivity techniques such as metagenomic sequencing to improve diagnostic sensitivity.

## Conclusion

LP CSF demonstrates higher sensitivity than EVD samples for early diagnosis of CNSIs in post-craniotomy patients with indwelling EVD. In clinical practice, when EVD results are negative but there is high clinical suspicion of CNSIs, concurrent LP should be performed for further confirmation.

## Data Availability

The raw data supporting the conclusions of this article will be made available by the authors, without undue reservation.
